# Effect of oral glucocorticoid intake on autonomic cardiovascular control

**DOI:** 10.1186/s40064-015-1378-8

**Published:** 2015-10-19

**Authors:** F. Cottin, V. Malcurat, H. Zorgati, F. Prieur, Z. Labsy, M. C. Do, O. Gagey, K. Collomp

**Affiliations:** CIAMS, Université Paris-Sud, Université Paris-Saclay, 91405 Orsay Cedex, France; CIAMS, Université d’Orléans, 45067 Orléans, France; Ecole supérieure d’ostéopathie, ESO Paris SUPOSTEO, Champs sur Marne, France; Département des analyses, AFLD, Châtenay-Malabry, France

**Keywords:** Blood pressure, Baroreflex, Time frequency analysis, Prednisone, Doping

## Abstract

This study analyzed baroreflex sensitivity, heart rate and systolic blood pressure variabilities during an oral 1 week administration of prednisone. This study examined the hypothesis that prednisone might change both systolic blood pressure level and baroreflex sensitivity. Twelve physically active male subjects participated to a double-blind, randomized cross-over study consisting of two 1-week periods of treatment separated by a 4-week drug-free washout period: placebo (PLA) or prednisone (PRED). Trials were performed by each subject four times on the second (D2) and seventh (D7) day of each treatment period. ECG and blood pressure were continuously recorded to compute heart rate variability, systolic blood pressure variability and baroreflex sensitivity components with the smoothed pseudo Wigner Ville distribution and baroreflex analysis. Following D2 prednisone treatment, both HR (PLA: 60.8 ± 10.5 vs. PRED: 65.8 ± 9.1 beats min^−1^, p = 0.008) and low frequency component of systolic blood pressure variability (D2: 3.09 ± 0.19 vs. D7: 2.34 ± 0.19, p < 0.041) increased whereas other components did not change. Over 7 days of treatment, LF-SBP amplitude increased (D2: 2.71 ± 0.89 vs. D7: 3.87 ± 0.6 mmHg, p = 0.037). A slight increase in both HR and LF-SBPV were observed suggesting a potential sympathetic cardiovascular stimulus. Although we found a significant effect of the 1-week prednisone treatment on heart rate and low frequency power of systolic blood pressure variability, we reported neither an increase in the systolic blood pressure level nor a decrease in the baroreflex sensitivity. Therefore, the fragility of our results cannot support a deleterious effect of 1-week administration of prednisone on the autonomic cardiovascular control which might be involved in cardiovascular diseases.

## Background

A few days of glucocorticoid intake has been shown to improve performance during submaximal exercise (Le Panse et al. 2009; Collomp et al. 2008; Arlettaz et al. 2007). Thus, under current World Anti-Doping Agency (WADA) legislation, all glucocorticoids are prohibited in-competition when administered systemically, although other routes of administration, topical or local, are permitted.

Glucocorticoid treatment entails significant cardiovascular effects. Indeed, chronic intake of synthetic glucocorticoids like prednisolone, methylprednisolone, triamcinolone or dexamethasone has been shown to increase blood pressure level (Whitworth et al. 1989; Brotman et al. 2006). Conversely, glucocorticoid deficiency is associated with low blood pressure and vascular sensitivity to hormonal changes (Grunfeld and Eloy 1987). In addition, the fludrocortisone is a glucocorticoid traditionally used as a treatment for hypotension (Ong et al. 2013; Waeber and Pruvot 2013). However, discrepant results reported that corticosterone treatment for 4–6 days in control rats had no significant effect on mean arterial pressure and HR (Scheuer and Bechtold 2002). Other authors also confirmed the lack of increase in systolic blood pressure in humans after administration of dexamethasone (a synthetic glucocorticoid with a higher activity than prednisone) at low dose (1 mg/day) and high dose (3 mg/day) during 4 days (Marquet et al. 1999).

Different mechanisms might account for an increase in blood pressure. Indeed, cortisol administration (Connell et al. 1987) has been reported to increase plasma volume, extracellular fluid volume, exchangeable sodium and body weight. An increase in renal vascular resistances might be involved, although calculated total peripheral resistances are unchanged (Connell et al. 1987). Direct and indirect indexes of sympathetic activity were either unchanged or suppressed during cortisol administration, suggesting that cortisol-induced hypertension is not mediated by an increase in sympathetic tone (Kelly et al. 1998).

Overall, glucocorticoid excess is associated with hypertension in man and in animals (Grunfeld and Eloy 1987; Pirpiris et al. 1993). Steroids-induced hypertension is independent of salt intake and can be prevented by glucocorticoid antagonists, such as RU486. Conversely, Grünfeld suggested that in some conditions, endogenous glucocorticoids contribute to the maintenance of blood pressure by enhancing vascular sensitivity to hormonal stimuli.

Accordingly, studies in animals showed that systemic elevations in glucocorticoids can modulate the arterial baroreflex effectiveness (Gardiner and Bennett 1983). Indeed, systemic administration of corticosterone modulates baroreflex control of both renal sympathetic nerve activity and heart rate, resulting in an increase in the arterial pressure midpoint and decrease in the baroreflex sensitivity, independently of changes in arterial pressure in rats (Scheuer and Mifflin 2001; Scheuer and Bechtold 2002). These results in animal models support the hypothesis that glucocorticoid administration in human might decrease the baroreflex sensitivity. Although the relationships between glucocorticoids and cardiovascular diseases are complex, physiological changes induced by glucocorticoid intake, if confirmed, might precipitate some cardiovascular diseases (Whitworth et al. 1995).

Analyses of heart rate variability (HRV), blood pressure variability (SBPV) and baroreflex sensitivity (BRS) have been used to assess the autonomic cardiovascular control, to predict cardiovascular morbidity (La Rovere et al. 2001; Robinson et al. 2003) and to evaluate overall cardiovascular health (Camm et al. 1996). As a matter of interest, baroreflex gain is usually estimated by administration of vasoactive drugs (Pagani et al. 1988) or pressure stimuli to the subject’s carotid baroreceptors with a neck collar (Ogoh et al. 2005). With these methods, substantial SBP and HR variabilities can be observed. Porta et al. (2000) suggested to remain cautious about these procedures, since they are based on non-physiological stimuli that may alter the actual baroreflex gain. Several techniques have been proposed to evaluate the baroreflex gain based on the spontaneous variability of SBP and HR. Among them, the spectral method allowed to investigate the spontaneous interactions between HRV and SBPV with no any other physiological or pharmacological maneuvers (Cottin et al. 2008; Porta et al. 2000). Therefore, this method should be particularly adequate to test the effect of a pharmacologic treatment on the baroreflex sensitivity such as glucocorticoid administration for instance.

To our knowledge, no study has investigated the effects of a 1-week administration of prednisone on the spontaneous baroreflex sensitivity. The aim of the present work was to analyze HRV, SBPV and BRS during a 1-week oral administration of glucocorticoids, in order to confirm the hypothesis that steroids can modify both systolic blood pressure level and BRS.

## Methods

Twelve healthy male volunteers (age: 20.8 ± 1.2 years; weight: 73.3 ± 10.7 kg; height: 178.5 ± 7.3 cm) were enrolled in the study. Before measurements, participants were familiarized with the experimental procedure and informed of the risks associated with the protocol. Ethics committee approval (No. 11-015, UFR STAPS Orsay, France) and written informed consent were obtained including the authorization to publish their data. This study was a part of a larger research protocol with blood samples, biomechanical measures (EMG, forceplate), local oxygen uptake measures (NIRS) and cardiovascular measures. Therefore, the protocol was drastically formatted and financed. To achieve all the measures we planned, only 12 subjects could have been involved. Only male subjects were involved to avoid the possibility of menstrual hormone interferences. They did not take any glucocorticoid or non-steroidal anti-inflammatory drug for at least 6 months prior to the study. Subjects were asked to maintain their exercise routines, normal food intake and were required to avoid competitive strenuous situations, any caffeine and alcohol for 24 h prior to each experimental session.

The double-blind, randomized cross-over study consisted of two 1-week periods of treatment for each subject separated by a 4-week drug-free washout period: placebo (PLA) or prednisone (PRED). PLA (gelatin) and PRED (trade name: Cortancyl 20 mg, tablets, Sanofi-Aventis Laboratory, Paris) were packaged in identical capsules. During the experimental periods, the subjects took three capsules per day of either PLA or PRED (60 mg) at home, between 7 and 8 am, over 7 days. They were questioned about the treatment they thought they had received first and were unable to report any difference. In addition, to ensure that they exactly follow the prescription, subjects had to fill in a form each time they took their treatment. Each subject came four times at the hospital on the second (PLA2 or PRED 2, acute effect) and seventh (PLA7 or PRED7, long-term effect) day of each treatment period, 3 h after the capsules ingestion.

### Data collection procedures

ECG was digitized and recorded with a PowerLab device (ADInstruments Ltd, AUS) with a sampling frequency of 1000 Hz. Beat to beat RR intervals were extracted from ECG using Chart7 pro soft (Chart7 pro, ADInstruments, AUS). Extrasystoles were manually processed by averaging the short and long RR intervals respectively preceding and following the extrasystole.

A Finometer device (TNO, BMI, The Netherlands) was used to record blood pressure from a cuff placed on a middle finger. BP signal yielded by the Finometer device has been previously experimentally validated during laboratory tests (Idema et al. 1989; Imholz et al. 1991). The Finometer was connected to the PowerLab that digitized and sampled BP signal at 1000 Hz. The series of systolic blood pressure (SBP) were assessed using a detection technique provided with the Chart7 pro software.

Signals were recorded in a quiet room at the hospital at a constant temperature (22 °C). The subjects were supine and their breathing frequency was paced at 0.25 Hz during 10 min via audio feedback.

The subjects had at least 20 min of rest in a supine position before cardiovascular measures.

### Signal processing

#### Time frequency analysis

The Smoothed Pseudo Wigner–Ville Distribution (SPWVD) was used to compute instantaneous components of HRV and SBP variability. It was performed with the cardiovascular toolbox developed in the scientific environment SCILAB (INRIA, France). The SPWVD provides a continuous evaluation of the amplitude and frequency, giving a nearly “instantaneous” complex FFT spectrum for each beat, with a high resolution achieved by independent time and frequency smoothing. Then, according to the standards (Camm et al. 1996), the instantaneous time frequency components were computed in low (LF-RR and LF-SBP from 0.04 to 0.15 Hz) and high frequency (HF-RR and HF-SBP from 0.15 to 0.4 HZ) bands of SBP variability and HRV. The spectral power was computed in LF and HF ranges (spectral components) by integrating the power spectral density (PSD) as following:

$$ LF = \sum\limits_{f = 0.04}^{0.15} {PSD \cdot \varDelta f\quad {\text{and}}\quad HF = \sum\limits_{f = 0.15}^{0.4} {PSD \cdot \varDelta f\;({\text{ms}}^{ 2} \;{\text{or}}\;{\text{mm}}^{ 2} {\text{Hg}})} } . $$

#### Baroreflex sensitivity (BRS)

The baroreflex sensitivity was assessed in LF (LF-BRS) and HF (HF-BRS) bands at rest. The spectral baroreflex sensitivity (BRS) is supported by the hypothesis of a linear relation between the input (BP) and output (RR) of the model. The degree of linearity between the two signals is estimated by the value of the coherence function. Ranging from 0 to 1, it was accepted that RR and SBP spectra had a reliable linear relationship when the coherence index was higher than 0.5 (Taylor and Eckberg 1996; de Boer et al. 1985). Next, the averaged spectral gain in HF and LF bands was the modulus of the transfer function between RR and SBP spectra (Maestri et al. 1998; Mangin et al. 2001; Monti et al. 2002).

### Statistics

Normality of data was examined by Kolmogorov–Smirnov test. When the data distribution was normal the differences between treatments (PLA vs. PRED) and treatment duration (D2 vs. D7) were tested with a two way ANOVA for repeated measures (Sigmastat 3.5, Systat software inc., UK). All pairwise multiple comparison procedures are given with the Holm-Sidak method. When the data distribution was not normal, an ANOVA on ranks was used. A p < 0.05 was considered as significant.

## Results

### Treatment effect (PRED vs. PLA)

HR increased following prednisone treatment (PLA: 60.8 ± 10.5 vs. PRED: 65.8 ± 9.1 beats min^−1^, p < 0.05, Table 1) and the power of performed test with α = 0.05 was 0.822. However, absolute (LFRR and HFRR) and normalised (LFnu, HF nu and LF/HF ratio) components of HRV were not different between PLA and PRED in both LF and HF bands (Table 1).

**Table 1:** Combined effects of the prednisone treatment (placebo vs. prednisone) and the treatment duration (Day 2 vs. Day 7) on heart rate (HR) and HR variability, expressed in absolute (low frequency: LFRR and high frequency amplitude: HFRR) and normalized (LFnu, HFnu and LF/HF) components

	Placebo		Prednisone	
	Day 2	Day 7	Day 2	Day 7
HR (bpm)	59.0 ± 8.4^1^	62.7 ± 12.3	66.9 ± 9.2	64.8 ± 9.4
LFRR (ms)	34.0 ± 8.6	30.8 ± 7.7	31.9 ± 17.2	30.8 ± 13.8
HFRR (ms)	57.0 ± 33.7	49.4 ± 38.1	44.5 ± 32.4	49.5 ± 35.9
LFnu	0.41 ± 0.12	0.45 ± 0.14	0.45 ± 0.10	0.42 ± 0.12
HFnu	0.59 ± 0.12	0.55 ± 0.14	0.55 ± 0.10	0.58 ± 0.12
LF/HF ratio	0.76 ± 0.41	0.90 ± 0.47	0.87 ± 0.29	0.80 ± 0.37

LFnu = LFRR/(LFRR + HFRR) and HFnu = HFRR/(LFRR + HFRR). Data are expressed as mean ± SD

^1^ *P* < 0.05 vs prednisone

SBP (PLA: 108 ± 10 vs. PRED: 106 ± 14 mmHg, NS, Table 2), DBP (PLA: 71 ± 7 vs. PRED: 71 ± 7 mmHg, NS, Table 2) and absolute SBPV components in the both LF and HF band did not change following prednisone administration (Table 2).

**Table 2:** Combined effects of the prednisone treatment (placebo vs. prednisone) and the treatment duration (Day 2 vs. Day 7) on systolic (SBP), diastolic (DBP), mean (MBP) blood pressure and SBP variability components: amplitude in low (LFSBP) and high frequency (HFSBP)

	Placebo		Prednisone	
	Day 2	Day 7	Day 2	Day 7
SBP (mmHg)	107 ± 12	109 ± 10	107 ± 15	105 ± 14
LFSBP (mmHg)	2.34 ± 0.51^1^	2.74 ± 1.50	3.08 ± 1.05^2^	3.99 ± 1.75
HFSBP (mmHg)	1.10 ± 0.47	1.17 ± 0.42	1.42 ± 0.38	1.24 ± 0.33

Data expressed as mean ± SD

^1^P < 0.05 vs prednisone

^2^P < 0.05 vs Day 7

BRS was not different between treatment conditions in both LF and HF band (Table 3).

**Table 3:** Combined effects of the prednisone treatment (placebo vs. prednisone) and the treatment duration (Day 2 vs. Day 7) on the baroreflex sensitivity (BRS) indexes

	Placebo		Prednisone	
	Day 2	Day 7	Day 2	Day 7
LF BRS gain (ms/mmHg)	15.32 ± 5.2	11.33 ± 7.3	11.07 ± 6.42	9.03 ± 5.19
LF Coherence	0.62 ± 0.06	0.63 ± 0.10	0.65 ± 0.06	0.67 ± 0.09
HF BRS gain (ms/mmHg)	43.58 ± 24.56	36.22 ± 24.09	29.24 ± 21.41	35.00 ± 25.24
HF coherence	0.70 ± 0.07	0.70 ± 0.05	073 ± 0.04	0.68 ± 0.03

Data are expressed as mean ± SD

There is no significant difference

### Duration of the treatment (D2 vs. D7)

HR, absolute and normalised HRV components did not change between D2 and D7 (Table 1).

SBP and HF-SBP amplitude did not change (Table 2) whereas LF-SBP amplitude increased between D2 and D7 (D2: 2.71 ± 0.89 vs. D7: 3.87 ± 0.6 mmHg, p = 0.037, Table 2) and the power of performed test with α = 0.05 was 0.499. On D2, post hoc statistics revealed that LF-SBP was higher with PRED than PLA (D2: 3.09 ± 0.19 vs. D7: 2.34 ± 0.19, p < 0.041).

BRS was not different between D2 and D7 in both LF and HF band (Table 3).

## Discussion

To our knowledge, the effects of 1-week administration of prednisone on spontaneous baroreflex sensitivity and cardiovascular variability in young subjects have never been investigated. The main results of this study is that 7 days of administration increased HR and the LF component of the SBPV whereas no change in SBP level was observed.

Regarding the SBP, the literature reports conflicting results about the effect of glucocorticoid treatment on systolic blood pressure; some studies observed an increase in SBP in rats (Scheuer and Bechtold 2002) and humans (Whitworth et al. 1989) whereas other did not (Marquet et al. 1999). In the present study, pharmacological treatment with the administration of 60 mg per day of prednisone during 7 days did not increase systolic blood pressure (SBP). This result is in accordance with a study by Marquet et al. (1999) who investigated the effect of dexamethasone (a synthetic glucocorticoid with a higher activity than prednisone) at low (1 mg/day) and high doses (3 mg/day) during 4 days on the cardiovascular system. As in our study, steroid administration did not change SBP whatever the dosage. Conversely, a significant increase in SBP was observed (Brotman et al. 2006) following higher doses of dexamethasone (6 mg/day for 5 days). According to correspondence tables for glucocorticoids (Meikle and Tyler 1977), 60 mg/day of prednisone would correspond to 9 mg/day of dexamethasone. Therefore, our dosage of prednisone should have produced stronger effects than the dexamethasone treatment administered in both of the above cited studies (Brotman et al. 2006; Marquet et al. 1999). In addition, differences in the methods used to measure blood pressure might account for this discrepancy. Indeed, BP results were the average of three punctual measurements in the two previous articles (Brotman et al. 2006; Marquet et al. 1999) whereas more accurate results are expected from the continuous, 10 min BP recording used in the present study.

The second interesting finding was that prednisone administration provoked both increase in HR and in LF-SBPV. The increase in HR could be due to a decrease in the vagal control or an increase in the sympathetic control of HR. The power of performed test with α = 0.05 was 0.822 suggesting the effect of the prednisone on HR was statistically strong. In this latter context, LF-SBP is only mediated by the sympathetic branch of the cardiovascular control. Therefore, the combination of this increase in both HR and LF-SBP suggests an increase in the sympathetic branch of the autonomic cardiovascular control (Pagani et al. 1986). Such an increase in resting condition might have deleterious effect in cardiac health (Camm et al. 1996; La Rovere et al. 2001). However, with a power of performed test with α = 0.05 was 0.499, the difference in LF-SBP was not very strong. In addition, the prednisone treatment did not provoke any increase in SBP and the above results were not in accordance with other studies (Brotman et al. 2006; Marquet et al. 1999) which found either a decrease or no change in HR. Therefore, it would be speculative to conclude that the treatment of prednisone entailed an increase in the sympathetic cardiovascular control which would have a deleterious effect on subject’s health.

Finally, a recent study has analyzed the spontaneous BRS during exhaustive dynamic exercise (Cottin et al. 2008) and it would worth investigating the effects of glucocorticoid intake on the BRS during this type of exercise in order to evaluate the benefit-risk ratio of such treatment.

## Conclusion

Seven days administration of glucocorticoids (prednisone 60 mg/day) did not change the systolic blood pressure level in young adults. However, a slight increase in both HR and LF-SBPV were observed suggesting a potential sympathetic cardiovascular stimulus. Although we found a significant effect of the 1-week prednisone treatment on heart rate and low frequency power of systolic blood pressure variability, we reported neither an increase in the systolic blood pressure level nor a decrease in the baroreflex sensitivity. Therefore, the fragility of our results cannot support a deleterious effect of 1-week administration of prednisone on the autonomic cardiovascular control which might be involved in cardiovascular diseases.

## Abbreviations

BP: blood pressure
BRS: baroreflex sensitivity
D2: day 2
D7: day 7
ECG: electrocardiogram
HF: high frequency
HFnu: HF-RR/(LF-RR + HF-RR)
HF-RR: high frequency power of HRV
HF-SBP: high frequency power of SBPV
HR: heart rate
HRV: heart rate variability
LF: low frequency
LF/HF: low to high frequency power ratio
LFnu: LF-RR/(LF-RR + HF-RR)
LF-RR: low frequency power of HRV
LF-SBP: low frequency power of SBPV
PLA: placebo
PRED: prednisone
RMSSD: root-mean-square differences of successive RR intervals
SBP: systolic blood pressure
SBPV: systolic blood pressure variability
SPWVD: Smoothed Pseudo Wigner–Ville Distribution
